# LONG-TERM GAIT SPEED TELEMONITORING IN OLDER ADULTS WITH MILD COGNITIVE IMPAIRMENT OR MILD DEMENTIA. THE DECI STUDY

**DOI:** 10.1093/geroni/igz038.1212

**Published:** 2019-11-08

**Authors:** Lorenzo M Donini, Alberto Rainoldi, Luca C Feletti, Gianluca Zia, Eleonora Poggiogalle, Susanna Del Signore, Monika Jurkeviciute on behalf of DECI Consortium

**Affiliations:** 1 Sapienza University, Rome, Italy; 2 University of Turin, Italy - Neuro Muscular Function Research group, School of Exercise and Sport Sciences, Department of Medical Sciences, Torino, Italy; 3 Caretek srl, Torino, Italy; 4 Bluecompanion LTD, London, England, United States; 5 Chalmers University of Technology, Gothenburg, Sweden

## Abstract

Non-intrusive telemonitoring of physical activity in Older Adults suffering from Mild Cognitive Impairment (MCI), or Mild Dementia (MD), was implemented as part of a 6-month multicomponent digital intervention in the DECI study (EU Horizon2020 grant No 643588). Methods: To estimate gait speed long-term trajectory, a processing algorithm was applied on individual accelerometry data continuously recorded via the ADAMO wrist-watch accelerometer. Speed Trend Analysis was performed if patients wore the device ≥90 days. Only outdoor activity was analyzed to reflect patients’ own natural gait speed. Only time spent in high or very-high-activity level is used, to eliminate rest periods (e.g. sitting on a bench, on a bus or driving). A raw mean walking speed was computed. Stride was computed from gender and height and walked distance from stride and step count. Mean walking speed was estimated by walking distance and duration. A rolling mean algorithm was applied to the computed mean 15-day baseline series, resulting in a new series representing normalized patient’s gait speed trajectory during the study. Results: Baseline characteristics: F/M=21/19; MCI/MD=36/4; age=75.4±6.0 years; BMI= 24.6±5,2; MMSE=26.5±2.4; education=8.9±4.0 years. Monitoring days=147±29. Overall three main patterns of gait speed trajectory were identified: “relative stability”, “improving trend” and “progressive decline”: No evident correlation with cognitive status was observed in the sample. Examples of individual patterns are shown. Conclusions: Gait Speed Analysis can describe physical function trajectory over time and identify decliners from stable or improving older adults. Further analyses may clarify the relationship between physical function changes and cognitive status.

